# A metadata framework for computational phenotypes

**DOI:** 10.1093/jamiaopen/ooad032

**Published:** 2023-05-09

**Authors:** Matthew Spotnitz, Nripendra Acharya, James J Cimino, Shawn Murphy, Bahram Namjou, Nancy Crimmins, Theresa Walunas, Cong Liu, David Crosslin, Barbara Benoit, Elisabeth Rosenthal, Jennifer A Pacheco, Anna Ostropolets, Harry Reyes Nieva, Jason S Patterson, Lauren R Richter, Tiffany J Callahan, Ahmed Elhussein, Chao Pang, Krzysztof Kiryluk, Jordan Nestor, Atlas Khan, Sumit Mohan, Evan Minty, Wendy Chung, Wei-Qi Wei, Karthik Natarajan, Chunhua Weng

**Affiliations:** Department of Biomedical Informatics, Vagelos College of Physicians & Surgeons, Columbia University Irving Medical Center, New York, New York, USA; Department of Biomedical Informatics, Vagelos College of Physicians & Surgeons, Columbia University Irving Medical Center, New York, New York, USA; Informatics Institute, Heersink School of Medicine, University of Alabama at Birmingham, Birmingham, Alabama, USA; Laboratory of Computer Science, Mass General Brigham, Boston, Massachusetts, USA; Department of Neurology, Mass General Brigham, Boston, Massachusetts, USA; Department of Pediatrics, Cincinnati Children’s Hospital Medical Center, Cincinnati, Ohio, USA; Department of Pediatrics, Cincinnati Children’s Hospital Medical Center, Cincinnati, Ohio, USA; Department of Medicine, Feinberg School of Medicine, Northwestern University, Chicago, Illinois, USA; Department of Biomedical Informatics, Vagelos College of Physicians & Surgeons, Columbia University Irving Medical Center, New York, New York, USA; Division of Biomedical Informatics and Genomics, Tulane University School of Medicine, New Orleans, Louisiana, USA; Department of Research Information Science & Computing, Mass General Brigham, Boston, Massachusetts, USA; Division of Genetics, University of Washington, Seattle, Washington, USA; Center for Genetic Medicine, Northwestern University, Chicago, Illinois, USA; Department of Biomedical Informatics, Vagelos College of Physicians & Surgeons, Columbia University Irving Medical Center, New York, New York, USA; Department of Biomedical Informatics, Vagelos College of Physicians & Surgeons, Columbia University Irving Medical Center, New York, New York, USA; Department of Biomedical Informatics, Vagelos College of Physicians & Surgeons, Columbia University Irving Medical Center, New York, New York, USA; Department of Biomedical Informatics, Vagelos College of Physicians & Surgeons, Columbia University Irving Medical Center, New York, New York, USA; Department of Biomedical Informatics, Vagelos College of Physicians & Surgeons, Columbia University Irving Medical Center, New York, New York, USA; Department of Biomedical Informatics, Vagelos College of Physicians & Surgeons, Columbia University Irving Medical Center, New York, New York, USA; Department of Biomedical Informatics, Vagelos College of Physicians & Surgeons, Columbia University Irving Medical Center, New York, New York, USA; Division of Nephrology, Department of Medicine, Vagelos College of Physicians & Surgeons, Columbia University Irving Medical Center, New York, New York, USA; Division of Nephrology, Department of Medicine, Vagelos College of Physicians & Surgeons, Columbia University Irving Medical Center, New York, New York, USA; Division of Nephrology, Department of Medicine, Vagelos College of Physicians & Surgeons, Columbia University Irving Medical Center, New York, New York, USA; Division of Nephrology, Department of Medicine, Vagelos College of Physicians & Surgeons, Columbia University Irving Medical Center, New York, New York, USA; Department of Epidemiology, Columbia University Mailman School of Public Health, New York, New York, USA; Department of Medicine, University of Calgary, Calgary, Alberta, Canada; Department of Pediatrics, Vagelos College of Physicians & Surgeons, Columbia University Irving Medical Center, New York, New York, USA; Department of Biomedical Informatics, Vanderbilt University, Nashville, Tennessee, USA; Department of Biomedical Informatics, Vagelos College of Physicians & Surgeons, Columbia University Irving Medical Center, New York, New York, USA; Department of Biomedical Informatics, Vagelos College of Physicians & Surgeons, Columbia University Irving Medical Center, New York, New York, USA

**Keywords:** electronic health records, phenotype, metadata

## Abstract

With the burgeoning development of computational phenotypes, it is increasingly difficult to identify the right phenotype for the right tasks. This study uses a mixed-methods approach to develop and evaluate a novel metadata framework for retrieval of and reusing computational phenotypes. Twenty active phenotyping researchers from 2 large research networks, Electronic Medical Records and Genomics and Observational Health Data Sciences and Informatics, were recruited to suggest metadata elements. Once consensus was reached on 39 metadata elements, 47 new researchers were surveyed to evaluate the utility of the metadata framework. The survey consisted of 5-Likert multiple-choice questions and open-ended questions. Two more researchers were asked to use the metadata framework to annotate 8 type-2 diabetes mellitus phenotypes. More than 90% of the survey respondents rated metadata elements regarding phenotype definition and validation methods and metrics positively with a score of 4 or 5. Both researchers completed annotation of each phenotype within 60 min. Our thematic analysis of the narrative feedback indicates that the metadata framework was effective in capturing rich and explicit descriptions and enabling the search for phenotypes, compliance with data standards, and comprehensive validation metrics. Current limitations were its complexity for data collection and the entailed human costs.

## INTRODUCTION

Computational phenotypes, which consist of clinical concept representations and programming logic, are essential to scale precision medicine and observational clinical studies. Multiple repositories have been developed to curate phenotypes and support their retrieval and reuse.^1–3^ The Phenotype Knowledge Base (PheKB) is one early example.^4^ Similarly, the Observational Health Data Sciences and Informatics (OHDSI) community, the Million Veterans Program, and the *All of Us* program, and the health data research UK community have each created phenotype libraries.^5–7^

Computational phenotypes vary significantly in their knowledge representations, documentation, and underlying data models.^8^ The existing phenotype descriptions often consist of a combination of free text and flow diagrams that are readable mainly by humans, not necessarily by machines, causing significant barriers for phenotype reuse, portability, and reproducibility. Subsequently, many computational phenotypes have been created redundantly due to difficulty with reusing existing ones. For example, there are more than a dozen type-2 diabetes mellitus (DM) phenotypes that use different inclusion and exclusion criteria, logic, code sets, and data domains—the name alone (eg, “diabetes”) fails to convey their differences. Implementing those phenotypes on the same EHR database retrieved only partially overlapping or different patient cohorts.^9^ This problem is further complicated by the fact that computational phenotypes are designed for different use cases. For example, one diabetes phenotype may be optimized for characterizing treatment pathways, whereas another may have better sensitivity for case identification.

More comprehensive and explicitly defined phenotype metadata promises to enable phenotype retrieval, reuse, and sharing. Some efforts have been made to create metadata for computational phenotypes to provide information needed for algorithm implementation. One set of desiderata, Mo et al. suggested ways to standardize computable representations of phenotype algorithms.^5^ A second set of desiderata, Chapman et al. suggested standards for the infrastructure of a phenotype library to improve portability and retrieval,^10^ comprising 14 suggestions about modeling, logging, validation, and sharing and warehousing of phenotypes. It did not, however, comment on metadata structure. Similar publications have proposed methods to improve the searchability, retrievability or machine readability of phenotype library frameworks.^8^^,^^11^ However, few studies have focused directly on metadata explicitness or phenotype validation methods and metrics. Therefore, we propose a novel metadata framework that is designed to be more accessible to phenotype users and includes comprehensive contextual knowledge about phenotype definitions, algorithms, performance metrics, and limitations. Our work is intended to facilitate task-oriented phenotype reuse by diverse stakeholders and improve the rigor and reproducibility of observational healthcare research.

## METHODS

We employed a mixed methods approach combining working group meetings, pilot annotation, and 5-point Likert question surveys to develop the metadata framework. Our study was performed as part of broader Electronic Medical Records and Genomics (eMERGE) network research activities.^12^ Participation of each of the eMERGE sites was approved by their respective IRBs. The framework consists of metadata elements that serve a prompts for annotation, such as “What is the phenotype definition?,” “What was the data source (i.e. EHR, Claims)?,” and “What method was used for validating the phenotype (i.e. chart review)?”

### Working group meetings

The eMERGE Network had an existing phenotyping working group of phenotyping experts with varying experiences from medical centers geographically distributed in the United States. We participated in those biweekly meetings from March to December, 2022, and discussed metadata as part of the agenda. Our goal was to make a framework of metadata criteria that could be used to annotate phenotypes by researchers both within eMERGE and beyond. In a typical meeting, we had brainstorming sessions on what metadata should be part of the framework which we then compiled into a document and discussed. The metadata framework was updated based on the group feedback iteratively.

### Framework refinement and test annotation

We identified 39 metadata elements based on group consensus. The subsections of the framework were Background Information, Algorithm Specifics, Performance Metrics, and Limitations. We annotated diabetes phenotypes as a sample test case for the framework. Two reviewers (MS and NA) performed a pilot annotation of 8 type-2 DM phenotypes, 3 of which were in the PheKB library and 5 of which were presented in a prior publication.^4^ One reviewer (MS) was a member of eMERGE.

### Survey

We used online third-party software (Qualtrics, Provo, UT) to create a survey that prompted participants to describe their background and experience with computable phenotypes and to rate the usefulness of each metadata element on a scale of 1–5, with 1 being the least useful and 5 being the most useful. We also used open-ended questions (Supplementary Appendix #2) to elicit narrative feedback.

We used e-mail and community forum posts to recruit survey participants from the eMERGE phenotype working group (https://phekb.org/phenotyping-groups/emerge-phenotype-wg), the OHDSI (https://www.ohdsi.org/) community, the Department of Biomedical Informatics at Columbia University (https://www.dbmi.columbia.edu/), the National COVID Cohort Collaborative (N3C, https://ncats.nih.gov/n3c), and the Monarch Initiative (https://monarchinitiative.org/).

### Qualitative analysis

We conducted a thematic analysis of the free-text survey responses. One author (MS) did the coding and thematic analysis on free text questions that had responses by at least 20% of survey participants with shared themes were expressed by at least 2 respondents.

### Statistical analysis

We tallied the multiple choice question ratings and performed subgroup analysis by stratifying the score distributions into 2 groups to determine if the responses differed between clinicians (defined as respondents with formal postgraduate clinical training at the level of intern or higher) and non-clinicians using Mann–Whitney U-tests, using R version 4.1.1.

## RESULTS

The 2 annotators filled out the metadata framework on all type-2 DM phenotypes to completion. Both annotators filled out the framework on each phenotype in fewer than 60 min. They discussed and resolved any discrepancies. Elements of the phenotype metadata framework are shown in Table 1.

**Table 1.** Elements of the phenotyping data framework

**Background information**
What is the phenotype definition?
Does the phenotype definition specify what patients will be identified, anyone currently or previously with the phenotype or newly diagnosed with the phenotype?
Does the definition specify the clinical setting for phenotype diagnosis (ie, inpatient)?
How has the phenotype been adopted?
When was the phenotype last updated?
Corresponding author contact information
Prior publication Pubmed ID
Has the phenotype been published in a phenotype library?
Did the investigators have clinical expertise?
Did the investigators have informatics expertise?
**Phenotype algorithm**
What was the data source (ie, EHR, claims)?
Were the source data structured (ie, CDM)?
Were the source data semi-structured (ie, problem list)?
Were the source data unstructured (ie, free text)?
Were the source data grouped by terminologies (ie, ICD-09/10)?
What data domains were used in the phenotype (ie, conditions, procedures)?
Was the phenotype rule based?
Was the phenotype machine learning based?
Was the phenotype natural language processing based?
Did the algorithm identify subtypes of the phenotype?
**Phenotype performance**
What method was used for validating the phenotype (ie, chart review)?
What was the validation population?
What was the phenotype prevalence?
What were the validation guidelines?
What was the definition of the validation phenotype?
Sensitivity
Specificity
Negative predictive value (NPV)
Positive predictive value (PPV)
Did most patients fulfill the phenotype criteria at similar points in their disease course?
Did most patients who fulfilled the phenotype criteria have similar disease presentations?
Did patients with new (incident) cases of the disease fulfill the phenotype criteria?
Did patients with chronic (prevalent) cases of the disease fulfill the phenotype criteria?
Polygenic score (PGS)
Other
**Limitations**
Did the phenotype lose a substantial amount information from the source data?
Can the phenotype be generalized to many populations other than the source and/or validation populations?
How do you envision using the phenotype (ie, clinical trial recruitment, clinical or public health study, translational or genetic study, clinical decision support)?
Other

CDM: common data model; EHR: electronic health record; ICD: International Classification of Diseases; NPV: negative predictive value; PPV: positive predictive value; PGS: polygenic score.

A total of 39 people provided complete responses. Background information on these survey respondents is shown in Supplementary Appendix #1, Table S1. The tabulated distribution of phenotype metadata element ratings is shown in Supplementary Appendix #1, Tables S2–S5.

**Figure 1.**
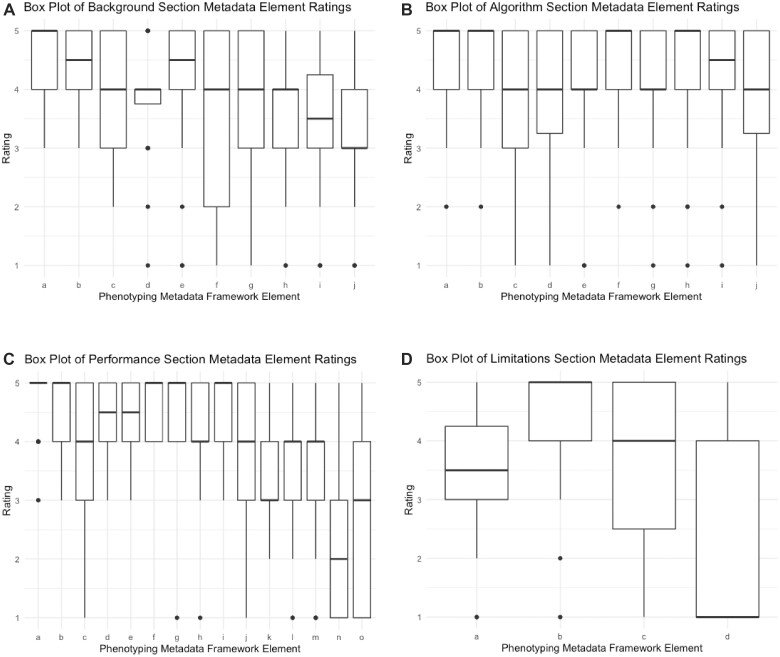
(A) Box plot of background section metadata element ratings. a = What is the phenotype definition?; b = Does the phenotype definition specify what patients will be identified, anyone currently or previously with the phenotype or newly diagnosed with the phenotype?; c = Does the definition specify the clinical setting for phenotype diagnosis (ie, inpatient)?; d = How has the phenotype been adopted?; e = When was the phenotype last updated?; f = Corresponding author contact information; g = Prior publication Pubmed ID; h = Has the phenotype been published in a phenotype library?; i = Did the investigators have clinical expertise?; j = Did the investigators have informatics expertise? (B) Box plot of algorithm section metadata element ratings. a = What was the data source (ie, EHR, claims)?; b = Were the source data structured (ie, CDM)?; c = Were the source data semi-structured (ie, problem list)?; d = Were the source data unstructured (ie, free text)?; e = Were the source data grouped by terminologies (ie, ICD-09/10)?; f = What data domains were used in the phenotype (ie, conditions, procedures)?; g = Was the phenotype rule based?; h = Was the phenotype machine learning based?; i = Was the phenotype natural language processing based?; j = Did the algorithm identify subtypes of the phenotype? (C) Box plot of performance section metadata element ratings. a = What method was used for validating the phenotype (ie, chart review)?; b = What was the validation population?; c = What was the phenotype prevalence?; d = What were the validation guidelines?; e = What was the definition of the validation phenotype?; f = sensitivity; g = specificity; h = negative predictive value (NPV); i = positive predictive value (PPV); j = Did most patients fulfill the phenotype criteria at similar points in their disease course?; k = Did most patients who fulfilled the phenotype criteria have similar disease presentations?; l = Did patients with new (incident) cases of the disease fulfill the phenotype criteria?; m = Did patients with chronic (prevalent) cases of the disease fulfill the phenotype criteria?; n = polygenic score (PGS); o = Other. (D) Box plot of limitations section metadata element ratings. a = Did the phenotype lose a substantial amount information from the source data?; b = Can the phenotype be generalized to many populations other than the source and/or validation populations?; c = How do you envision using the phenotype (ie, clinical trial recruitment, clinical or public health study, translational or genetic study, clinical decision support)?; d = Other.

Box plots of responses to survey questions regarding the Background Information, Algorithm, Performance, and Limitations sections are shown in Figure 1A–D. More than 90% of respondents assigned a rating of 4 or 5 to metadata framework elements concerning phenotype definition, validation method, validation population, sensitivity, and specificity.

Out of 39 respondents, 10 identified as being in the clinician subgroup and 14 identified as being in the non-clinician subgroup. We found no statistically significant differences in responses between the 2 subgroups.

The results of the thematic analysis regarding the strengths and limitations of the phenotyping metadata framework are shown in Table 2.

**Table 2.** Thematic analysis of questions regarding the phenotyping metadata framework strengths and limitations

What are the strengths of the phenotyping metadata framework? (*n* = 16)	What are the limitations of the phenotyping metadata framework? (*n* = 11)
Descriptiveness or transparency (*n* = 7)	Too detailed or complex (*n* = 5)
Phenotype standards (*n* = 5)	Challenging to keep it updated (*n* = 2)
Performance or validation metrics (*n* = 3)	
Searchability or retrievability (*n* = 3)	

*Note*: The top rows (bold) show the original questions and the number of respondents. The other rows show common themes and the number of respondents who expressed each theme.

A total of 14 participants responded to the question “Do you feel that the phenotyping metadata framework will lead to more consistent use of phenotypes by investigators?” Of those, 9 responses were affirmative, 4 were ambivalent, and 1 response did not answer the question directly. The full thematic analysis is shown in Supplementary Appendix #2.

## DISCUSSION

To our knowledge, this study reports the first effort to develop a metadata framework for computational phenotypes. We envision that phenotype developers could use this framework to annotate metadata efficiently. The metadata represented in our framework has the potential to support reproducible and potentially automatable ways to help clinical researchers determine which phenotype is best for a specific use case. Our evaluation shows many metadata framework elements that are considered useful. The metadata elements on algorithm performance were considered a strength, possibly due to the transparency and explicitness of such metrics for enabling easy selection of computational phenotypes. Furthermore, the respondents considered the standardization and rich description of computational phenotypes to be important metadata. We found no preference differences between clinicians and non-clinicians when rating the importance of metadata elements.

Our study is limited by subjectivity in the design of the survey instrument. A Delphi method may have been more rigorous than the methods we used. Furthermore, our working group discussions were performed without a guide. However, we believe the rich, extensive discussions forming the basis for the instrument and the large number of respondents provided ample opportunity for multiple opinions to be heard.

Our survey respondents may not represent all users of computational phenotypes. However, we did reach out to multiple organizations and channels that comprise a large number of clinical researchers. Our intent was to gauge a general sense of whether our metadata framework would help experts annotate phenotypes and we believe that our methodology, the robust response, and the consistency of those responses demonstrate that enumerating standardized, useful metadata elements is feasible.

Updates to the metadata framework are needed, based on the results of this analysis, such as fewer non-specific prompts (eg, “other”) or questions about phenotype limitations, as well as additional elements to support machine-learning-based phenotypes and allow for more unstructured responses. Future research should explore structuring the framework responses to maximize retrievability using a combination of free text, semi-structured, and unstructured responses. We expect that structured responses may be useful for empirical metrics such as sensitivity and specificity, whereas semi-structured or free text responses may be more appropriate for the other elements, such as phenotype definition. The framework may also benefit from addition of a data dictionary to help standardize responses. Based on our experience to date, we believe that our data-driven approach will improve the consistency and quality of computable phenotypes.

## CONCLUSIONS

Using a mixed-methods approach, we have developed a comprehensive framework for defining computational clinical phenotypes. Use of this framework may help better curate patient data used for both observational and prospective healthcare research.

## Supplementary Material

ooad032_Supplementary_Data

## Data Availability

The data underlying this article are available in the article and in its Supplementary Material.
