# Supplementary material for: A metadata framework for computational phenotypes
Source: JAMIA Open. 2023 May 9;6(2):ooad032. doi: 10.1093/jamiaopen/ooad032 (PMC10168627; doi:10.1093/jamiaopen/ooad032)
Supplement: ooad032_Supplementary_Data [file ooad032_supplementary_data.docx]

**Appendix #1: Tabulated Survey Results**

Table 1: Background information of survey respondents

| **Criteria** | **N(%)** |
| --- | --- |
| **Extent of Clinical Training** |  |
| Clinical Fellow or Attending Physician | 8 (33.33) |
| Intern or Resident | 2 (8.33) |
| Medical School | 6 (25.00) |
| No Formal Training | 8 (33.33) |
| **Extent of Informatics Training** |  |
| Doctoral Degree or Postdoctoral Scientist | 11 (45.83) |
| Master's Degree | 12 (50.00) |
| No Formal Training | 1 (4.17) |
| **Years in Observational Healthcare Research** |  |
| More than 5 Years | 14 (58.33) |
| Between 3 and 5 Years | 6 (25.00) |
| Between 1 and 3 Years | 3 (12.5) |
| Less Than 1 Year | 1 (4.17) |
| **Number of Phenotypes Used Previously** |  |
| Between 201 and 500 | 1 (4.17) |
| Between 101 and 200 | 3 (12.5) |
| Between 51 and 100 | 7 (29.17) |
| Between 10 and 50 | 6 (25) |
| Fewer than 10 | 7 (29.17) |
| **Publications to Date** |  |
| More than 50 | 13 (54.17) |
| Between 21 and 50 | 5 (20.83) |
| Between 6 and 10 | 1 (4.17) |
| Between 1 and 5 | 5 (20.83) |
| **Primary Use of Phenotypes** |  |
| Clinical or Public Health Research | 17 (70.83) |
| Genetic or Translational Research | 6 (25) |
| Other | 1 (4.17) |

Table 2: Rating of the background section of phenotyping metadata framework

| **Question, (N, %)** | **1** | **2** | **3** | **4** | **5** | **Total** |
| --- | --- | --- | --- | --- | --- | --- |
| What Is The Phenotype Definition? | 0 (0.00) | 0 (0.00) | 2 (8.33) | 5 (20.83) | 17 (70.83) | 24 (100) |
| Does The Phenotype Definition Specify What Patients Will Be Identified, Anyone Currently Or Previously With The Phenotype Or Newly Diagnosed With The Phenotype? | 0 (0.00) | 0 (0.00) | 4 (16.67) | 8 (33.33) | 12 (50.00) | 24 (100) |
| Does The Definition Specify The Clinical Setting For Phenotype Diagnosis (i.e. Inpatient)? | 0 (0.00) | 1 (4.17) | 8 (33.33) | 8 (33.33) | 7 (29.17) | 24 (100) |
| How Has the Phenotype Been Adopted? | 1 (4.17) | 1 (4.17) | 4 (16.67) | 13 (54.17) | 5 (20.83) | 24 (100) |
| When Was The Phenotype Last Updated? | 2 (8.33) | 1 (4.17) | 2 (8.33) | 7 (29.17) | 12 (50.00) | 24 (100) |
| Corresponding Author Contact Information | 4 (16.67) | 3 (12.50) | 4 (16.67) | 5 (20.83) | 8 (33.33) | 24 (100) |
| Prior Publication Pubmed ID | 2 (8.33) | 0 (0.00) | 5 (20.83) | 8 (33.33) | 9 (37.50) | 24 (100) |
| Has The Phenotype Been Published In A Phenotype Library? | 2 (8.33) | 3 (12.50) | 3 (12.50) | 13 (54.17) | 3 (12.50) | 24 (100) |
| Did The Investigators Have Clinical Expertise? | 2 (8.33) | 1 (4.17) | 9 (37.50) | 9 (25.00) | 6 (25.00) | 24 (100) |
| Did The Investigators Have Informatics Expertise? | 2 (8.33) | 2 (8.33) | 9 (37.50) | 9 (29.17) | 4 (16.67) | 24 (100) |

Table 3: Rating of the algorithm section of phenotyping metadata framework

| **Question, N(%)** | **1** | **2** | **3** | **4** | **5** | **Total** |
| --- | --- | --- | --- | --- | --- | --- |
| What Was The Data Source (i.e. EHR, Claims)? | 0 (0.00) | 1 (4.55) | 2 (9.09) | 5 (22.73) | 14 (63.64) | 22 (100) |
| Were The Source Data Structured (i.e. CDM)? | 0 (0.00) | 1 (4.55) | 2 (9.09) | 6 (27.27) | 13 (59.09) | 22 (100) |
| Were The Source Data Semi-Structured (i.e. Problem List)? | 1 (4.55) | 2 (9.09) | 4 (18.18) | 6 (27.27) | 9 (40.91) | 22 (100) |
| Were The Source Data Unstructured (i.e. Free Text)? | 1 (4.55) | 2 (9.09) | 3 (13.64) | 8 (36.36) | 8 (36.36) | 22 (100) |
| Were The Source Data Grouped By Terminologies (i.e. ICD-09/10)? | 2 (9.52) | 0 (0.00) | 2 (9.52) | 7 (33.33) | 10 (47.62) | 21 (100) |
| What Data Domains Were Used In The Phenotype (i.e. Conditions, Procedures)? | 0 (0.00) | 1 (4.55) | 2 (9.09) | 6 (27.27) | 13 (59.09) | 22 (100) |
| Was The Phenotype Rule Based? | 1 (4.76) | 1 (4.76) | 2 (9.52) | 7 (33.33) | 10 (47.62) | 21 (100) |
| Was The Phenotype Machine Learning Based? | 1 (4.55) | 2 (9.09) | 2 (9.09) | 5 (22.73) | 12 (54.55) | 22 (100) |
| Was The Phenotype Natural Language Processing Based? | 1 (4.55) | 1 (4.55) | 3 (13.64) | 6 (27.27) | 11 (50.00) | 22 (100) |
| Did The Algorithm Identify Subtypes Of The Phenotype? | 1 (4.55) | 0 (0.00) | 5 (22.73) | 6 (27.27) | 11 (45.45) | 22 (100) |

Table 4: Rating of the performance section of phenotyping metadata framework

| **Question, N(%)** | **1** | **2** | **3** | **4** | **5** | **Total** |
| --- | --- | --- | --- | --- | --- | --- |
| What Method Was Used For Validating The Phenotype (i.e. Chart Review)? | 0 (0.00) | 0 (0.00) | 1 (4.76) | 4 (19.05) | 16 (76.19) | 21 (100) |
| What Was The Validation Population? | 0 (0.00) | 0 (0.00) | 2 (10.00) | 6 (30.00) | 12 (60.00) | 20 (100) |
| What Was The Phenotype Prevalence? | 1 (4.76) | 0 (0.00) | 8 (38.10) | 3 (14.29) | 9 (42.86) | 21 (100) |
| What Were The Validation Guidelines? | 0 (0.00) | 0 (0.00) | 3 (15.00) | 7 (35.00) | 10 (50.00) | 20 (100) |
| What Was The Definition Of The Validation Phenotype? | 0 (0.00) | 0 (0.00) | 2 (10.00) | 8 (40.00) | 10 (50.00) | 20 (100) |
| Sensitivity | 0 (0.00) | 0 (0.00) | 0 (0.00) | 8 (38.10) | 13 (61.90) | 21 (100) |
| Specificity | 1 (4.76) | 0 (0.00) | 0 (0.00) | 8 (38.10) | 12 (57.14) | 21 (100) |
| Negative Predictive Value (NPV) | 1 (4.76) | 0 (0.00) | 4 (19.05) | 8 (38.10) | 8 (38.10) | 21 (100) |
| Positive Predictive Value (PPV) | 0 (0.00) | 0 (0.00) | 3 (14.29) | 6 (28.57) | 12 (57.14) | 21 (100) |
| Did Most Patients Fulfill The Phenotype Criteria At Similar Points In Their Disease Course? | 1 (4.76) | 1 (4.76) | 7 (33.33) | 5 (23.81) | 7 (33.33) | 21 (100) |
| Did Most Patients Who Fulfilled The Phenotype Criteria Have Similar Disease Presentations? | 0 (0.00) | 1 (4.76) | 10 (47.62) | 6 (28.57) | 4 (19.05) | 21 (100) |
| Did Patients With New (Incident) Cases Of The Disease Fulfill The Phenotype Criteria? | 2 (9.52) | 1 (4.76) | 5 (23.81) | 9 (42.86) | 4 (19.05) | 21 (100) |
| Did Patients With Chronic (Prevalent) Cases Of The Disease Fulfill The Phenotype Criteria? | 2 (9.52) | 2 (9.52) | 3 (14.29) | 10 (47.62) | 4 (19.05) | 21 (100) |
| Polygenic Score (PGS) | 6 (28.57) | 6 (28.57) | 5 (23.81) | 3 (14.29) | 1 (4.76) | 21 (100) |
| Other | 4 (30.77) | 2 (15.38) | 3 (23.08) | 2 (15.38) | 2 (15.38) | 13 (100) |

Table 5: Rating of the limitations section of phenotyping metadata framework

| **Question, (N, %)** | **1** | **2** | **3** | **4** | **5** | **Total** |
| --- | --- | --- | --- | --- | --- | --- |
| Did The Phenotype Lose A Substantial Amount Information From The Source Data? | 2 (10.00) | 2 (10.00) | 6 (30.00) | 5 (25.00) | 5 (25.00) | 20 (100) |
| Can The Phenotype Be Generalized To Many Populations Other Than The Source And/Or Validation Populations? | 1 (4.76) | 1 (4.76) | 3 (14.29) | 5 (23.81) | 11 (52.38) | 21 (100) |
| How Do You Envision Using The Phenotype (i.e. Clinical Trial Recruitment, Clinical or Public Health Study, Translational or Genetic Study, Clinical Decision Support)? | 3 (15.79) | 2 (10.53) | 4 (21.05) | 1 (5.26) | 9 (47.37) | 19 (100) |
| Other | 6 (54.55) | 0 (0.00) | 1 (9.09) | 2 (18.18) | 2 (18.18) | 11 (100) |

**Appendix #2: Thematic Analysis of Free-Text Questions**

**“What are the strengths of the phenotyping metadata framework?”**

**Phenotype Metadata Strengths (n = 16):**

-Concrete criteria

-It looks like a comprehensive description of a phenotype

-The most important feature the metadata framework should provide is to filter out current phenotypes. In addition, it can serve as a guideline for future phenotype publication.

-clarity about phenotypes and improved power by combining studies

-ensures appropriate and accurate application while allow recognition of potential limitations.

-performance tracking and complexity

-easy search. standardized phenotype format. information for validation, or if there is validation

-avoid duplication of phenotyping similar or the same conditions. Knowing the generalizability or validity of a phenotype would give confidence to using the same definition for a different purpose

-Great way to really understand observational health research and its limitations

-Structured format to clearly assess phenotypes and how they are used

-Help quickly find the right phenotype to use

-It should be as transparent as possible about the inputs to and validation process of the phenotype.

-i don't know what the phenotyping metadata framework is

-I think that the primary strengths are the explicit specification of a standard set of information that everyone can rely on when considering using an existing phenotype or creating a new one. This information not only increases the trust in an existing resource but also increases the likelihood of others creating useful phenotypes when creating new ones.

-transparent documentation of phenotype processing

-I think that a phenotyping metadata framework will help in generating higher-quality phenotype data, leading to more trustworthy results from research studies.

-***Descriptiveness or transparency***

-It looks like a comprehensive description of a phenotype

-Structured format to clearly assess phenotypes and how they are used

-clarity about phenotypes and improved power by combining studies

-Great way to really understand observational health research and its limitations

-I think that the primary strengths are the explicit specification of a standard set of information that everyone can rely on when considering using an existing phenotype or creating a new one. This information not only increases the trust in an existing resource but also increases the likelihood of others creating useful phenotypes when creating new ones.

-avoid duplication of phenotyping similar or the same conditions. Knowing the generalizability or validity of a phenotype would give confidence to using the same definition for a different purpose

-Concrete criteria

***-Performance or validation metrics***

-performance tracking and complexity

-easy search. standardized phenotype format. information for validation, or if there is validation

-transparent documentation of phenotype processing

***-Phenotype standards***

-I think that the primary strengths are the explicit specification of a standard set of information that everyone can rely on when considering using an existing phenotype or creating a new one. This information not only increases the trust in an existing resource but also increases the likelihood of others creating useful phenotypes when creating new ones.

-I think that a phenotyping metadata framework will help in generating higher-quality phenotype data, leading to more trustworthy results from research studies.

-easy search. standardized phenotype format. information for validation, or if there is validation

-ensures appropriate and accurate application while allow recognition of potential limitations.

-The most important feature the metadata framework should provide is to filter out current phenotypes. In addition, it can serve as a guideline for future phenotype publication.

***-Searchability or retrievability***

-easy search. standardized phenotype format. information for validation, or if there is validation

-The most important feature the metadata framework should provide is to filter out current phenotypes. In addition, it can serve as a guideline for future phenotype publication.

-Help quickly find the right phenotype to use

**What are the limitations of the phenotyping metadata framework?**

**Phenotype Metadata Framework Limitations (N=11):**

-It is not clear how the fields will be populated (e.g. how patient presentations would be described or how generalizability you other populations would be established).

-It seems like a huge engineering work to build this metadata for all existing phenotypes.

-I'm unclear of the origins of these metadata elements. Was there a process followed to come up with these? Should there be (beyond this survey instrument)? i.e. a literature review, some kind of delphi round (or other qualitative methods - not an expert in that).

-lost of information

-how to keep it update? how often will you do it?

-not that I can think of. Can always tweak an existing phenotype for a particular unique situation, but having a starting point would save a lot of time

-Might not be able to answer all questions adequately, leading to perhaps stalled research?

-potentially complex leading to people not filling it

-Perhaps not an explicit limitation, but there is probably a fine line where providing enough information to satisfy one person results in too much information to someone else. I think finding a sweet spot of providing the right amount and type of information is really important.

-Variation in data sources (EHR, Claims) Health system variations, phenotype use case variations, variations in validation procedures.

-Lack of examples, e.g., for "Phenotype Name" in "PhenotypingAlgorithm", how would I select which phenotype name to use? For example, for "Granulomatosis with polyangiitis", should I use the more commonly used name "Wegener's granulomatosis" or?

***-Too detailed or complex***

-Might not be able to answer all questions adequately, leading to perhaps stalled research?

-Potentially complex leading to people not filling it

-Perhaps not an explicit limitation, but there is probably a fine line where providing enough information to satisfy one person results in too much information to someone else. I think finding a sweet spot of providing the right amount and type of information is really important.

-It is not clear how the fields will be populated (e.g. how patient presentations would be described or how generalizability you other populations would be established).

***-Challenging to keep it updated***

-It seems like a huge engineering work to build this metadata for all existing phenotypes.

-how to keep it update? how often will you do it?

**Do you feel that the phenotyping metadata framework will lead to more consistent use of phenotypes by investigators?**

**Phenotype metadata framework consistency of use (n=14)**

-If authors are required to fill the metadata sheet, it may prompt them to think more deeply about their phenotypes, ie facilitate development. In phenotype re-use, having such information will be useful. Not clear if phenotypes will be re-used according to the information provided in metadata through.

-Yes. In the ideal case, the future publication should use this framework as a guideline to better inform readers and provide a much more reusable resources.

-yes

-yes - if it remains easy to access, identify etc.

-yes

-yes, i believe so.

-Yes, definitely

-Ideally!

-if used correctly, yes

-Yes definitely

-Absolutely, not only more consistent use but also more reliable, reproducible, and portable information as well.

-One can hope.

-Yes

-i don't know what the phenotyping metadata framework is

Metadata Framework Helpfulness Themes

***Affirmative (n=9)***

-Yes. In the ideal case, the future publication should use this framework as a guideline to better inform readers and provide a much more reusable resources.

-yes

-yes - if it remains easy to access, identify etc.

-yes

-yes, i believe so.

-Yes, definitely

-Yes definitely

-Absolutely, not only more consistent use but also more reliable, reproducible, and portable information as well.

***Ambivalent (n=4)***

-If authors are required to fill the metadata sheet, it may prompt them to think more deeply about their phenotypes, ie facilitate development. In phenotype re-use, having such information will be useful. Not clear if phenotypes will be re-used according to the information provided in metadata through.

-Ideally!

-One can hope.

-if used correctly, yes
